# A Manual of Auscultation and Percussion

**Published:** 1849-10

**Authors:** 


Art. VIII.—*A Manual of Auscultation and Percussion.* By M. Barth, Agrégé to the Faculty of Medicine at Paris, &c., &c., and M. Henry Roger, Physician to the Bureau Central of the Parisian Hospitals, &c. &c. Translated, with additions, by Francis G. Smith, M. D., Lecturer on Physiology in the Philadelphia Association for Medical Instruction, one of the Editors of the Medical Examiner, &c., &c., 8vo. pp. 167. Second edition. Philadelphia: Lindsay & Blakiston. 1849.

We are pleased to find that this very valuable work has reached the second edition. We well remember with what advantage we consulted the original, while pursuing the studies of which it treats, in Paris. The translator, Dr. Smith, has corrected, in the present edition, the few errors that escaped him in the first, and has introduced such additional matter, as was demanded by the existing state of the science of auscultation and percussion. "As a short manual of the most important diseases of the chest and abdomen, and their method of diagnosis by physical signs," we are acquainted with no work that possesses stronger claims to the confidence of the profession than the one before us. A condensed manual on the subject has been much and long needed, by beginners in the study of auscultation and percussion, and while this work is of the description alluded to, and addressed particularly to this class of readers, it at the same time will form a pleasant and serviceable companion for the practitioner. As we remarked in a notice of the first edition, it is one of the most esteemed of the text books used by the Parisian medical student, and its principal author, Dr. Barth, is one of the most skilful of living auscultators. All of the above works are for sale in this city at the bookstore of Messrs. Beckwith & Morton.

